# Pathological Changes That Follow Section of the Sciatic Nerve

**Published:** 1850-06

**Authors:** 


*Pathological Changes that Follow Section of the Sciatic Nerve.*—In the same number of the British and Foreign Medical Chirurgical Review, M. Brown Séquard calls in question the inference that certain patholigical changes, which follow the section of the sciatic nerve, show the dependence of the nutritive operations upon nervous agency. The following facts and reasonings, seem to be conclusive:

"M. Séquard found that section of the nerve in frogs produced no pathological change whatever, save the atrophy consequent upon the paralysis, except when water found its way into the wound. He then divided the nerve in a number of rabbits and guinea-pigs, and placed some of the animals at liberty in a room with a paved floor, whilst he confined others in a box, whose bottom was thickly covered with bran and hay. In a fortnight, the former set exhibited an obvious disordered action in the paralyzed limbs; the claws were entirely lost, the extremities of the feet were swollen, and the exposed tissues were red, engorged, and covered with fleshy granulations. At the end of a month, these alterations were more decided; and a necrosis had supervened in the denuded bones. On the other hand, in the animals confined in the boxes, no such injuries had accrued. It is obvious, therefore, that these changes do not proceed directly from section of the nerve; but that they are consequent upon the friction and compression to which the paralyzed members are subject against a hard soil, in consequence of the inability of the animal to feel or avoid it."

